# The Toxicological Risk Assessment of Cu, Mn, and Zn as Essential Elemental Impurities in Herbal Medicinal Products with Valerian Root (*Valeriana officinalis L.*, radix) Available in Polish Pharmacies

**DOI:** 10.1007/s12011-021-02779-y

**Published:** 2021-06-09

**Authors:** Kamil Jurowski, Maria Fołta, Barbara Tatar, Mehmet Berkoz, Mirosław Krośniak

**Affiliations:** 1 Institute of Medical Studies, Medical College, Rzeszów University, Al. mjr. W. Kopisto 2a , 35-959 Rzeszów, Poland; 2 Department of Food Chemistry and Nutrition, Medical College, Jagiellonian University, Medyczna 9, 30-688 Kraków, Poland; 3 Department of Biochemistry, Van Yuzuncu Yil University, Faculty of Pharmacy, 65080 Van, Turkey

**Keywords:** Valerian root, Elemental impurities, Herbal medicinal product, ICH Q3D, PDE, Toxicological analysis, Risk assessment

## Abstract

The toxicological risk assessment (TRA) of elemental impurities (EI) in especially herbal medicinal products (HMP) is a significant challenge for pharmaceutical industry. In Europe, very popular are traditional HMP with valerian root (*Valeriana officinalis* L., radix) for relief of mild symptoms of mental stress and to aid sleep. The aim of our unique article is the comprehensive TRA of Cu, Mn, and Zn as EI in HMP with *V. officinalis* L., radix available in Polish pharmacies. This article is a continuation of our previously conducted studies about TRA of heavy metals (Pb and Cd) in these same samples. Investigated elements were determined by flame atomic absorption spectrometry (F AAS). The values of the correlation coefficients (R > 0.998) confirm the linearity of the applied instrument for precision and accuracy of results. The recoveries, LOD and LOQ values were acceptable. Our results show that all investigated HMP with valerian root available in Polish pharmacies contain Cu (0.16–0.23 mg/L), Mn (0.11–0.76 mg/L), and Zn (0.22–0.48 mg/L) at a very low level. Based on our estimation of EI including single dose (µg/20 mL) and estimated daily intake (µg/day), our results confirm the safety of all pharmaceuticals. To the best of our knowledge, the Cu, Mn, and Zn impurity profile in HMP with *V. officinalis* L., radix is described for the first time. The applied methodology and results are extremely important from regulatory toxicology point of view (ICH Q3D elemental impurities guideline for pharmaceuticals).

**Supplementary Information:**

The online version contains supplementary material available at 10.1007/s12011-021-02779-y.

## Introduction

**Fig. 1:**
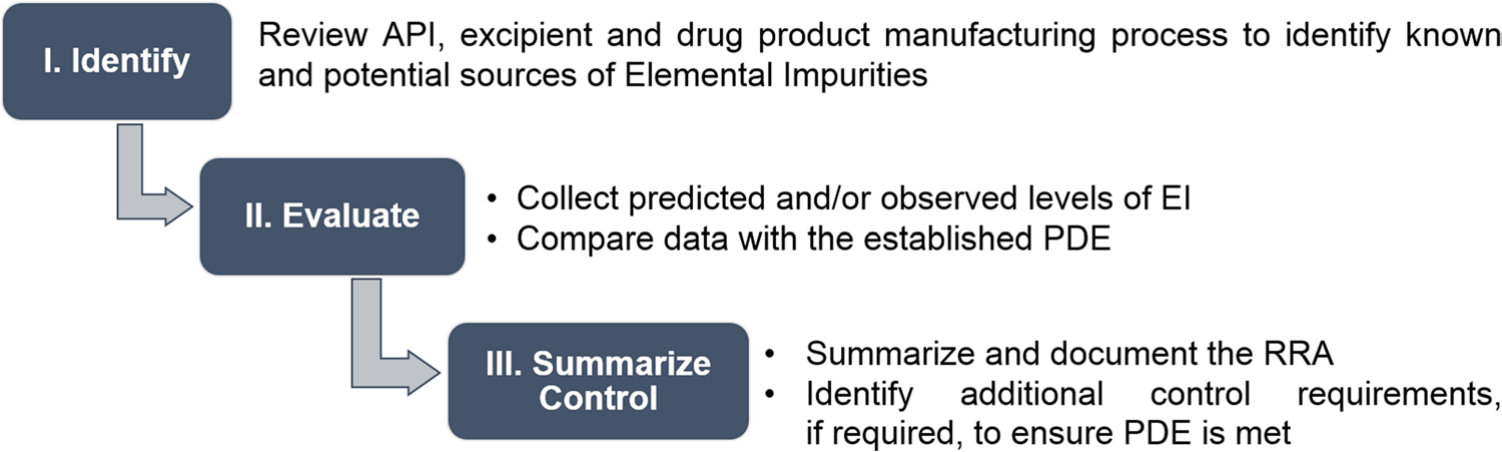
Briefly summary of the safety assessment steps of EI proposed by ICH Q3D guideline [2]

The toxicological safety assessment of elemental impurities (EI) in plants and herbs as raw materials applied in the pharmaceutical industry is a very important task, but unfortunately it is also a unique topic of scientific articles. Considering additionally the fact that EI do not provide any therapeutic benefit to the patient, their contents in the final pharmaceutical/drug products are extremely important from a toxicological point of view and should be controlled within acceptable limits [1]. The main document about EI in pharmaceuticals is ICH Q3D guideline (International Council for Harmonization of Technical Requirements for Pharmaceuticals for Human Use) as guidelines about acceptable limits and proposed safety assessment approaches [2]. In general, the safety assessment of EI in final pharmaceutical products described in ICH Q3D guideline consists of three important steps (Fig. 1).

The first step is review of active pharmaceutical ingredients (API) and pharmaceutical manufacturing process to identify known and potential sources of EI. It should be noted that there exist several sources of known EI, especially [3]: residual catalysts (added intentionally during synthesis) and metal reagent residues. On the other hand, there exist also potential sources of EI [2, 4] like impurities through interactions with processing equipment or container/closure systems and ingredients of the pharmaceutical/drug product. The last source is crucial when herbal medicinal products (HMP) are considered, due to the fact that herbs can uptake EI from different routes [5]. The next step is evaluation of obtained results with acceptable limits. Hence, in this step the measured levels of EI in analyzed product are compared with permitted daily exposure (PDE) values for each element (based on ICH Q3D guideline [1, 2]). The last step is summary of toxicological risk assessment (TRA) based on earlier conducted evaluation process.

As was mentioned earlier, an interesting, but rare field of study is the toxicological safety assessment of EI in HMP. HMP can be defined as “any medicinal product, exclusively containing as active ingredients one or more herbal substances or one or more herbal preparations, or one or more such herbal substances in combination with one or more such herbal preparations” [6]. Among this kind of pharmaceutical products, a very popular herb in Europe applied as API is *Valeriana officinalis* L. (valerian root) [7]. It should be noted that valerian root is one of the major herbs processed by the Polish pharmaceutical industry, with a total annual harvest exceeding 1000 tones in Poland [8].

In many countries (especially in Europe), *V. officinalis* L. as tincture is a very popular traditional HMP for relief of mild symptoms of mental stress and to aid sleep [9]. It should be underlined that orally administered dry extracts of valerian root prepared with ethanol (tincture) in the recommended dosage (approximately 20 mL as single dose) have been shown to improve sleep latency and sleep quality. However, it is well documented that different conditions of valerian root cultivation may cause crucial changes in the chemical composition of this herb and also its medical effects [10]. Hence, due to HMP, it is extremely important to control the content of elements accumulated as EI during the plant’s growth [11]. In general, there are two main uptake routes for EI in herbs: (1) EI uptake from soil (roots) and (2) metal uptake from air (dry and wet deposition).

It is well known that the herb’s element content stemming from root uptake is affected not only by the geochemical characteristics of soil in which they grow, but additionally by the ability of herbs to selectively accumulate certain elements [12]. On the other hand, uptake EI also enter herbs via dry and wet deposition (air and rain water) [13]. Because the subject of our research is the root of *V. officinalis* L., radix, the element uptake from air may be omitted from our considerations. There is no doubt that all herbs require certain elements for growth and normal physiological functioning [14]. Because elements cannot be synthesized by the plants itself, hence uptake of elements from the environment is crucial [15]. Essential elements like copper, manganese, and zinc are important for herbs growth and physiological function; however, from a toxicological point of view, the excess of these elements can also exhibit potential harmful effects for patients [16].

Considering all described earlier facts, the aim of our article is the TRA of Cu, Mn, and Zn as EI in HMP with valerian root (*Valeriana officinalis* L., radix) available in Polish pharmacies. This article is a continuation of our previously conducted studies about TRA of heavy metals (Pb and Cd) in these same samples [17]. It should be noted that based on review of this essential topic in scientific literature, there are only a few professional articles about EI in HMP [18–23]. Hence to full fill this gap between science and industry, the contents of Cu, Mn, and Zn in samples (*n* = 5) of HMP with valerian root available in Polish pharmacies were determined by flame atomic absorption spectrometry (F AAS). To the best of our knowledge, the Cu, Mn, and Zn impurity profile as EI in HMP with *V. officinalis* L., radix is described for the first time. Our article is innovative due to the fact we included aspects of TRA in accordance with the ICH Q3D guideline, which is extremely important for regulatory purposes (pharmaceutical industry). Therefore, the greatest advantage of our research is focusing on the practical aspects of the applied approach. Additionally, we applied well validated analytical method and we confirm safety of HMP with valerian root available in Polish pharmacies. The disadvantage is the applied technique (F AAS) which is slower and more demanding in comparison to ICP-MS, however well validated.

## Materials and Methods

### Samples

In our research, we investigated all available in Polish Pharmacies HMP with valerian root (*V. officinalis* L., radix). Analyzed samples (*n* = 5) were herbal preparation in liquid dosage forms for oral use as tincture (ratio of valerian root to extraction solvent 1:5; extraction solvent: ethanol 60–80% (v/v)). For the best quality of methodological standards, the double-blind approach was applied. For this purpose, all samples were coded in random order (A–F). The brief characterization of the analyzed samples is shown in Table 1.

**Table 1:** The briefly characterization of investigated herbal medicinal products with valerian root (*Valeriana officinalis* L., radix) available in Polish pharmacies

Sample code	Ratio of valerian root to extraction solvent	Extraction solvent, concentration (v/v)	License
A	1:5	Ethanol, 60.0%	IL-0969/LN
B	1:5	Ethanol, 70.0%	14,040
C	1:5	Ethanol, 60.0%	IL-0025/LN
D	1:5	Ethanol, 80.0%	R/6696
E	1:5	Ethanol, 65.0%	IL-2663/LN

Due to the fact that all HMP were liquid samples (valerian tinctures), no additional sample preparation was needed. Hence, in situ determination was used (see “The Instrumentation and Determination of Cu, Mn, and Zn”).

### Chemicals

Demineralized water from a Milli-Q water purification system (Millipore, Bedford, MA, USA) was applied for the standard and sample preparation. Nitric acid (65%) for preparation of standard solutions was of spectroscopic grade (Merck SupraPur, Darmstadt, Germany).

Cu, Mn, and Zn standard stock solutions of 1.0 mg/L were obtained from Merck (Darmstadt, Germany). The applied certified reference material was Corn Flour (INCT-CF-3) obtained from the Institute of Nuclear Chemistry and Technology Department of Analytical Chemistry (Warsaw, Poland).

### The Instrumentation and Determination of Cu, Mn, and Zn

In most elemental analysis procedures, the first step before determination is homogenization and digestion of samples. However, due to the fact that all our HMP samples were liquid samples (valerian tinctures), this step was omitted (is not required). Hence, in situ analysis was applied.

The EI of copper, manganese, and zinc were determined using a Perkin-Elmer 5100 ZL atomic absorption spectrometer (Perkin-Elmer, Norwalk, CT, USA) with flame atomic absorption spectrometry (F AAS). For this purpose, appropriate elemental hollow cathode lamps were applied as the emission sources. Background corrections were conducted by Zeeman background correction approach. Applied similar analytical procedures were described in our early published articles [20, 21]. Detailed instrumental parameters are described in Supplementary materials 1 (SM1).

### The Safety Assessment of Cu, Mn, and Zn in Analyzed HMP

**Fig. 2:**
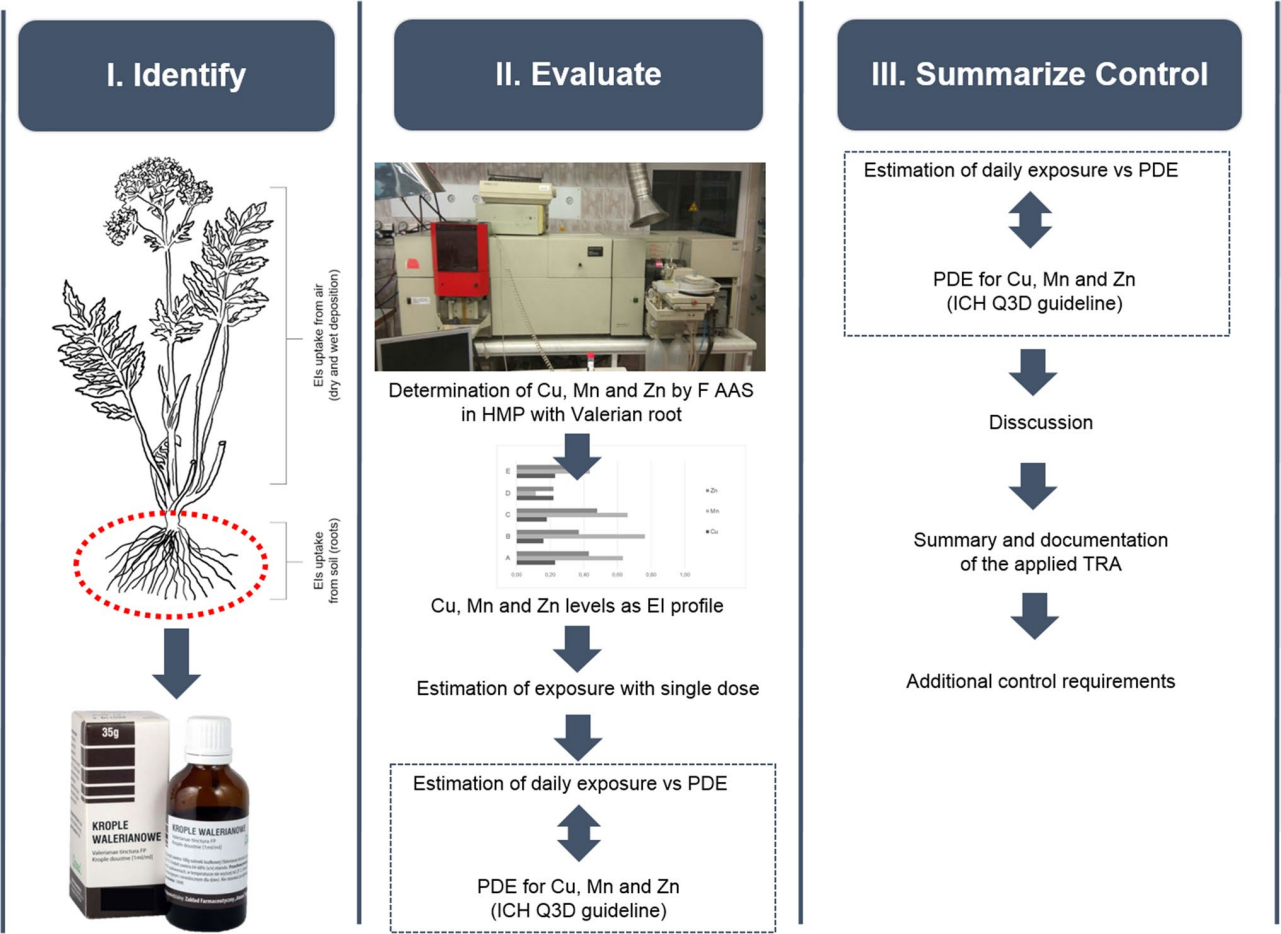
The schematic workflow of applied safety assessment of Cu, Mn and Zn as EI in samples of investigated HMP

The applied safety assessment approach consisted of three crucial steps in analogy to described earlier safety assessment steps of EI proposed by ICH Q3D guideline (see “Introduction”). The schematic idea of our research is shown in Fig. 2.

### Data Analysis

Obtained results were analyzed applying Educational Analysis Set SAS® 9 licensed by the Jagiellonian University in Krakow (initial analysis, not included). The results of five independent replicates were expressed as the mean ± standard deviation. Additionally, the descriptive statistics were made (minimum, maximum, mean, skewness, and kurtosis) using Origin 2021 Pro licensed by the Jagiellonian University in Krakow. Impurity profiles were plotted using Origin 2021 Pro licensed by the Jagiellonian University in Krakow.

## Results and Discussion

### The Essential Elements (Cu, Mn, and Zn) Impurity Profiles in HMP with *V. officinalis L.*, radix (Valerian Root)

**Fig. 3:**
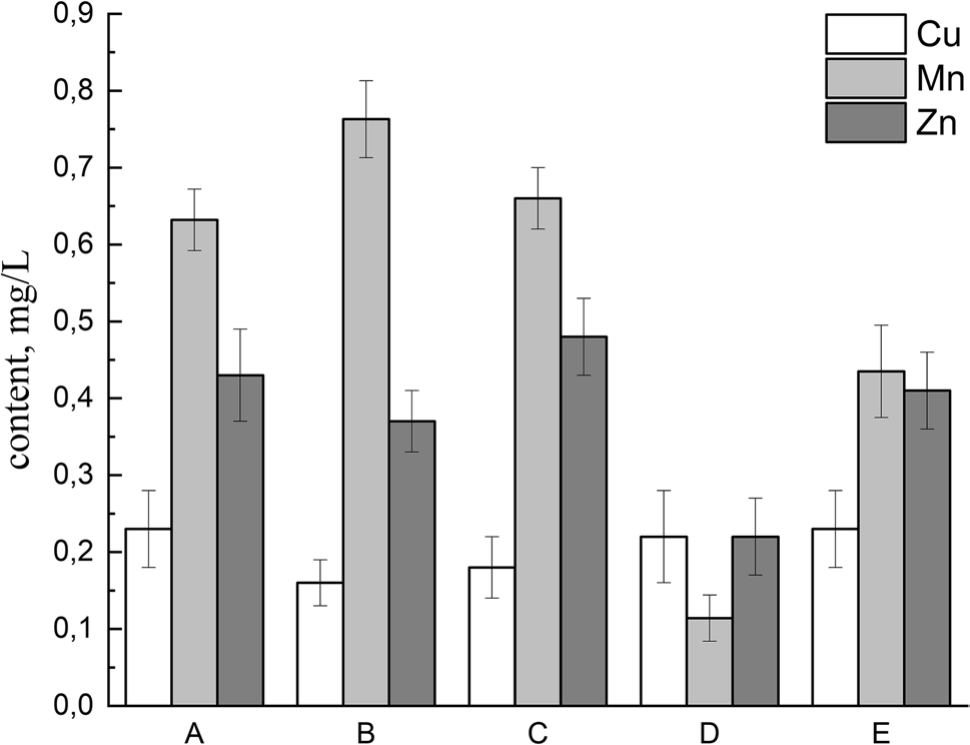
The essential elements’ (Cu, Mn, and Zn) impurity profile of investigated HMP (A, B, C, D, and E)

The EI profiles of investigated samples (*n* = 5; A–E) are presented in Fig. 3 as the essential elements’ impurity profiles (mg/L) determined in analyzed HMP available in Polish pharmacies. Additionally, the descriptive statistics of Cu, Mn, and Zn contents (minimum, maximum, and mean) in all samples is shortly described in Table 2.

**Table 2:** The descriptive statistics of Cu, Mn, and Zn levels in all analyzed samples of HMP with *V. officinalis* L., radix (valerian root) available in Polish pharmacies

Element	Minimum, mg/L	Maximum, mg/L	Mean, mg/L	Skewness	Kurtosis
Cu	0.16	0.23	0.20	1.35	1.95
Mn	0.11	0.76	0.52	0.45	− 1.35
Zn	0.22	0.48	0.38	0.89	1.95

In general, all investigated elements were present in all of the analyzed samples below 1.0 mg/L (in the range of 0.16 to 0.76 mg/L). The content of Cu was very similar in all investigated samples (in the range of 0.16 to 0.23 mg/L). On the other hand, the Mn concentration was quite similar—in the range of 0.11 to 0.76 mg/L. Additionally, the Zn content was also at similar range of 0.22 to 0.48 mg/L. The basic analysis of the overall content (Table 2) shows that Cu levels (mean = 0.20 mg/L) were approximately 2.5 times lower than Mn levels (mean = 0.52 mg/L), and Cu content was approximately 2 times lower than Zn levels (mean = 0.38 mg/L). Skewness and kurtosis values confirm the distribution of results and their consistency.

Individual content analysis shows the lowest content of Cu was in sample B (0.16 ± 0.02 mg/L) and the highest level was in sample A and E (approximately 0.23 ± 0.04 mg/L). On the other hand, the lowest level of Mn was in sample D (0.11 ± 0.05 mg/L) and the highest content was in sample B (0.76 ± 0.06 mg/L). Finally, the lowest level of Zn was in sample D (0.22 ± 0.06 mg/L) and the highest content was in sample C (0.48 ± 0.06 mg/L). Considering the concentration limits for copper in pharmaceuticals via oral route recommended by ICH Q3D guideline (300.0 μg/g [1, 2]), all of the investigated HMP with *V. officinalis L.*, radix meet the guidelines. Hence, our results confirm the safety of Cu levels in all samples. On the other hand, manganese and zinc are classified by ICH Q3D guideline as other metals, i.e., “elemental impurities for which PDEs have not been established due to their low inherent toxicity and/or differences in regional regulations which are not addressed in this guideline” 2]. In this situation, appropriate sources of information about acceptable levels of EI should be other guidelines and/or regional regulations. Based on review of scientific and regulatory literature, there is a lack of guidelines and/or regional regulations and practices related to Cu and Mn impurities. Hence, it is not possible to check or compare our results with any results. However, it can be summarized that the levels are very low (< 1.0 mg/L).

### The Estimation of Exposure of Investigated Essential Elemental Impurities HMP with *V. officinalis L.*, radix (Valerian Root)

The obtained results about impurity profiles in analyzed samples are crucial especially for other researchers about range of elemental impurities. The next step in the safety assessment approach is estimation of EI exposure for single-dose/one-time administration of the herbal medical product. For this purpose, appropriate calculations were made based on posology and method of administration described in monograph on *Valeriana officinalis* L., radix by European Medicine Agency (EMA/HMPC/150848/2015) [9]. Based on traditional use described in mentioned monograph, to aid sleep, a single dose (20 mL) should be administered half to 1 h before bedtime with an earlier dose during the evening if necessary [9]. The obtained results of Cu, Mn, and Zn impurities including the single dose of analyzed samples are shown in the first part of Table 3. This estimation is crucial for the final step of assessment of these elements’ exposure in daily intake of applied HMP.

**Table 3:** The estimated exposure of Cu, Mn, and Zn impurities to which the patient is exposed for single dose (µg/20 mL; first part of table) and daily dose (µg/day; second part of table) of the herbal medical product with *V. officinalis L.*, radix (valerian root) available in Polish pharmacies

Sample		Level, µg/20 mL			Level, µg/day		
		Cu	Mn	Zn	Cu	Mn	Zn
No	Code	Mean	Mean	Mean	Mean	Mean	Mean
1	A	4.60	12.64	8.60	13.80	37.92	25.80
2	B	3.20	15.26	7.40	9.60	45.78	22.20
3	C	3.60	13.20	9.60	10.80	39.60	28.80
4	D	4.40	2.28	4.40	13.20	6.84	13.20
5	E	4.60	8.70	820	13.80	26.10	24.60

Based on information in the monograph on *Valeriana officinalis* L., radix by European Medicine Agency (EMA/HMPC/150848/2015) [9], the frequency of application should be no more than three times per day. Hence, based on mentioned information and results from Table 3, the estimated daily exposure of Cu, Mn, and Zn impurities to which the patient is exposed for daily administration of the herbal medical product with *Valeriana officinalis* L., radix (Valerian root) is shown in the second part of Table 3.

The estimated daily exposure of Cu is relatively constant between analyzed samples (9.60–13.80 µg/day). Based on PDE value for Cu in pharmaceutical products (oral concentration) recommended by ICH Q3D guideline (3400 μg/day [2]), all of the samples meet the guidelines (each HMP is characterized by level of < 15 μg/day).

As was mentioned earlier, Mn and Zn are classified by ICH Q3D guideline as other metals [2]; hence, unfortunately, it is not possible to compare our results with PDE values or other published studies. Notwithstanding, estimated daily exposure of Mn and Zn impurities has significant value for other investigators and future works of ICH Q3D teams.

## Conclusions and Recommendations

Our results show that all investigated HMP with valerian root available in Polish pharmacies contain Cu (0.16–0.23 mg/L), Mn (0.11–0.76 mg/L), and Zn (0.22–0.48 mg/L) at a very low level (< 1 mg/L). All investigated pharmaceuticals meet the standards of ICH Q3D guideline due to copper levels. Comparison of obtained results for Mn and Zn impurities is not possible with PDE because ICH Q3D guideline defined these elements as other metals without any values for comparison. However, based on our estimation of EI including single dose (µg/20 mL) and estimated daily intake (µg/day), our results confirm the safety of all HMP. Hence, no additional control requirements are necessary. It can be summarized that each of the pharmaceuticals with valerian root (*Valeriana officinalis L.*, radix) for relief of mild symptoms of mental stress and to aid sleep available in Polish pharmacies does not represent a health hazard to the patients.

The advantages of our research are as follows: (1) practical methodology and (2) relevance of the obtained results from regulatory toxicology point of view (ICH Q3D elemental impurities guideline for pharmaceutical industry). The disadvantage is the applied technique (F AAS) which is slower and more demanding in comparison to ICP-MS.

Based on review of scientific literature, it would be important to carry out a broader TRA including other important metallic impurities and different herbal medicinal products containing *V. officinalis L.*, radix as in our last published articles [17, 24]. It should be underlined that based on last step of proposed safety assessment approach by ICH Q3D guideline, our simple but well documented TRA could be valuable for other researchers.

## Supplementary Information

Below is the link to the electronic supplementary material.

ESM 1 (DOCX 17 kb)

## Data Availability

All data generated or analyzed during this study are included in this published article and its supplementary information file.

## Abbreviations

AAS: Atomic absorption spectrometry technique
API: Active pharmaceutical ingredients
EI: Elemental impurities
F AAS: Flame atomic absorption spectrometry
HMP: Herbal medicinal product
ICH Q3D: International Council for Harmonization of Technical Requirements for Pharmaceuticals for Human Use
PDE: Permitted daily exposure
TRA: Toxicological risk assessment
