# Supplementary material for: The Toxicological Risk Assessment of Cu, Mn, and Zn as Essential Elemental Impurities in Herbal Medicinal Products with Valerian Root (Valeriana officinalis L., radix) Available in Polish Pharmacies
Source: Biol Trace Elem Res. 2021 Jun 9;200(4):1949–55. doi: 10.1007/s12011-021-02779-y (PMC8854135; doi:10.1007/s12011-021-02779-y)
Supplement: Supplementary file 1 — (DOCX 17 kb) [file 12011_2021_2779_MOESM1_ESM.docx]

Supplementary materials 1. Detailed instrumental parameters

The certified reference material was prepared from corn grown in Poland according to Polish standard PN-A-74205:1997. The material was sieved through the 250 μm nylon sieves and stored in a polyethylene (PE) bag. Approximately 50 kg of sieved corn flour was collected. Examination by optical microscopy revealed that Martin’s diameter of over 98% of particles was below 25 μm. The whole lot of corn flour was then homogenized by mixing for 20 hours in a 110 dm3 PE drum rotated in three directions. Preliminary homogeneity testing by XRF method and final checking of homogeneity by NAA after distribution of the material into containers revealed, that it is sufficiently homogeneous at least for a sample size ≥ 100 mg.

In order to assure the long-term stability, all containers with INCT-CF-3 were sterilized by electron beam radiation. Long-term stability was checked by analyzing concentrations of selected elements in the material stored in the air-conditioned room at 20 °C.

Short-term stability was examined by the determination of concentrations of the selected elements in the bottle stored in the CO2 incubator at 37 °C.

The shelf life of INCT-CF-3 has been established to be 31 December 2015.

The material was certified on the basis of a worldwide interlaboratory comparison, in which 92 laboratories from 19 countries. Analytical uncertainties and stability uncertainties were quantified to arrive at combined uncertainties of the certified values.

The comparison of certified values with measured values of determined elements is shown in Table S1.

**Table S1.** The comparison of certified values with measured values of determined elements.

| concentration, µg/g | certified values | measured values |
| --- | --- | --- |
| Cu | 1.63 ± 0.13 | 1.58 ± 0.18 |
| Mn | 4.98 ± 0.22 | 5.05 ± 0.18 |
| Zn | 20.09 ± 0.76 | 19.65 ± 0.78 |

The linear range of the calibration curve reached from the detection limit up to
0.0; 0.25; 0.5; 1.0; 2.0; 3.0 mg/L for Cu, 0.0; 0.25; 0.5; 1.0; 2.0; 5.0 mg/L for Mn, and 0.0; 0.25; 0.5; 1.0; 2.0; 3.0 for Zn mg/L, respectively. The values of correlation coefficients (R) are a good indicator of the linearity for AAS instrument for precision and accuracy of results, in our studies all correlation coefficients were acceptable (R > 0.998).

The limit of detection (LOD) was defined as (3 SD)/*a*, where SD is the standard deviation corresponding to 10 blank injections and “*a*” is the slope of the calibration function obtained for each microelement. The LODs were determined for Cu, Mn and Zn as 2.1 µg/L,
3.2 µg/L and 2.0 µg/L, respectively. On the other hand, LOQ is defined as (10 SD)/a, where SD is the standard deviation corresponding to ten blank injections and “a” is the slope of the calibration curve, obtained for each heavy metal.  The calculated LOQs were 6.5 µg/L for Cu,  10.3 µg/L for Mn and 6.2 µg/L for Zn. The recoveries obtained were acceptable: 96.8.0 % for Cu, 98.2 % for Mn and 97.6% for Zn.

Blank samples of ultrapure water were prepared applying the same procedure as for the samples to assess possible contamination during the sample preparation and analytical calibration step. All blank levels obtained were negligible. Newly prepared standard stock solutions were serially diluted and used to obtain calibration curves.

The detailed instrumental parameters are shown in table S2.

**Table S2.** Instrumental parameters for the determination of Cu, Mn and Zn.

| Operating parameters | essential trace elements | | |
| --- | --- | --- | --- |
|  | Cu | Mn | Zn |
| Wavelength [nm] | 324.8 | 279.5 | 213.9 |
| Lamp current [mA] | 15 | 15 | 18 |
| Slit width [nm] | 0.7 | 0.7 | 0.7 |
| Optimum working range [µg/kg] | 0.2-2.0 | 0.2-2.0 | 1.0-10.0 |
| Air [ L/min] | 9 | 10 | 10.8 |
| Acetylene [L/mn] | 2 | 2 | 2 |
